# Current practice, barriers and drivers to embedding environmental sustainability in undergraduate dental schools in the UK and Ireland

**DOI:** 10.1038/s41415-024-8011-6

**Published:** 2024-11-08

**Authors:** Jonathan Dixon, Nicolas Martin, James Field

**Affiliations:** 802900088891572731533 School of Clinical Dentistry, University of Sheffield, United Kingdom; 430814941065426480668 School of Dentistry, Cardiff University, Cardiff, United Kingdom

## Abstract

**Aims** This study aimed to: i) identify current teaching practice and approaches to embedding environmental sustainability (ES) in the undergraduate dental curriculum in the UK and Republic of Ireland (ROI); and ii) uncover existing barriers and drivers to incorporating ES in dental education.

**Methods** A questionnaire was developed and distributed to all dental schools in the UK and ROI in the form of an online survey. The intended respondents were deans, heads of schools, directors of education, or senior academics of all dental schools in the UK and ROI that deliver undergraduate dentistry/dental surgery programmes.

**Results** In total, 18 dental schools responded to the survey, representing a response rate of 100% from the intended respondents. Note 56% of dental schools do not currently teach ES. Time constraints and a lack of knowledge and learning resources were the most reported barriers.

**Conclusion** Currently, ES is not taught in most dental schools in the UK and ROI. Many schools face challenges in finalising the delivery modalities of ES teaching, particularly in clinical environments. Numerous barriers have been identified that complicate embedding this topic in the curriculum. Positively, universities, staff, students and the recently published learning outcomes are driving impactful change across the sector.

## Key points

- Environmental sustainability is an emerging topic in undergraduate dental curricula.
- There is significant support from universities, staff, students and regulators to embed environmental sustainability in the curriculum.
- Most dental schools are not currently teaching this topic; however, many have plans to do so after the recent General Dental Council learning outcomes update.
- Many schools are facing challenges in teaching this topic in clinical environments.

## Introduction

Environmental sustainability (ES) is a growing concern in dentistry, with research demonstrating that delivering oral healthcare, in its current form, is unsustainable.^1^^,^^2^^,^^3^^,^^4^^,^^5^ The most significant contributors to environmental impacts are patient travel and staff commute; procurement of equipment; instruments and dental materials; energy and water use; and waste disposal. Multiple healthcare organisations have set environmental goals, including the NHS in England and their commitment to achieve net zero by 2040.^6^ Achieving these goals will require a significant change in attitudes and behaviours across all healthcare professions.

Education at all levels of the oral health profession has been identified as a key strategic approach to deliver environmentally sustainable change.^4^^,^^7^ The Association for Dental Education in Europe (ADEE) have been leading the incorporation of ES in undergraduate education through a ‘Sustainability in Dentistry' special interest group, which has published two consensus reports.^8^^,^^9^ These papers both established the need to embed ES in dental education, and proposed learning outcomes and teaching and assessment methods specifically for ES. The FDI World Dental Federation has also been a key stakeholder in supporting ES change in the profession, with the publication of a consensus statement that brought together multiple partners from all sectors of the profession, including industry.^10^ This is supported by a ‘sustainability in dentistry' toolkit and infographic.^11^ Additionally, the FDI World Dental Federation have published an open access Massive Open Online Course (MOOC) that aims to educate the profession at all levels, from students to qualified professionals.^12^

The undergraduate dental curriculum must respond to emerging challenges, whether from societal pressures, new professional developments, or educational rationales and innovations. There is a strong desire from oral health professional students to include ES in their curriculum but with an element of nervousness that this should not provide additional time pressures or course workload.^13^^,^^14^^,^^15^^,^^16^^,^^17^ This is matched with significant support from academic staff.^8^^,^^9^^,^^13^^,^^16^^,^^17^ The inclusion of ES in the dental curriculum is now imminent in the UK, as the General Dental Council have adopted several learning outcomes published by the ADEE special interest group in the recent *The safe practitioner* framework.^18^ The General Dental Council have mandated compliance with the new framework for all graduates by 2030.

It is unclear if dental schools are currently teaching ES, with little evidence outlining current practice. It is, therefore, anticipated that some dental schools may be struggling to teach and assess this subject matter.^19^ Furthermore, a recent paper exploring a wide range of curriculum practices across Europe identified limited evidence of ES teaching in dental schools.^20^ Multiple surveys exploring academic staff and student opinion have been published and generally report very little experience of ES in the curriculum.^13^^,^^14^^,^^15^^,^^16^^,^^17^ Three of these studies also report some barriers that complicate the inclusion of ES in dental education; although, the limited response rate, number of institutions included and closed-ended questions are reported limitations.^13^^,^^16^^,^^17^

There is an appreciation that the evidence that supports our current understanding of the perceived challenges and the proposed solutions is derived from a relatively small number of higher education institutions. While there is a logical desire to generalise this body of knowledge across all institutions, we recognise that this is not necessarily correct and that there is a need to uncover existing practices and barriers across the UK and Republic of Ireland (ROI) as a whole. This study aimed to: i) identify current teaching practice and approaches to embedding ES in the undergraduate dental curriculum in the UK and ROI; and ii) uncover existing barriers that complicate, and drivers that facilitate, the inclusion of ES in dental education.

## Methodology

This study received ethical approval from the Dentistry Ethics Committee of the University of Sheffield (application number 056060). An eight-item questionnaire was developed and distributed to all dental schools in the UK and ROI in the form of an online survey (Google Form). The questions were conceptualised de novo by the authors due to an absence of previous research in the area. The question types and response options were informed by previous research. A range of multiple-choice, ‘yes/no' and free-text responses were used as appropriate to the question and construct asked (Table 1). The questions were separated into four sections:

**Table 1:** The survey questions and response options

Question	Response options
Please state the name of your institution and the country in which it is based	Free text
Does your dental school currently teach ES in the undergraduate curriculum?	Multiple choice (single selection): Yes, we have specific dedicated learning outcomes for ES Yes, but we don't have specific learning outcomes for ES Not yet but we have plans to do this soon No
How do you currently (or plan to) teach ES? Please select the approach that best matches your existing practice	Multiple choice (single selection): A standalone course for ES A single or group of lectures at one point in the programme ES forms part of our teaching in all years of our programme
Please detail the methods you use (or plan to use) to teach ES in non-clinical environments	Free text, no limits
Please detail the methods you use (or plan to use) to teach ES in clinical environments	Free text, no limits
What barriers do you face when planning to embed ES in your undergraduate curriculum?	Checkboxes (select all that apply) Lack of knowledge/expertise to teach ES in dentistry Time constraints/overloaded curriculum Lack of practical guidance to support me Limited learning resources for educators and students Challenges in assessing ES concepts Resistance from colleagues regarding the relevance of ES in dentistry Limited evidence base for ES in dentistry Conflict with regulations, policy and healthcare system
Do you face any additional barriers that are not listed in the previous question?	Free text, no limits
Please list the drivers/facilitators that have supported your approach or plans to embed environmental sustainability in your undergraduate curriculum	Free text, no limits

- Responding institution
- Current teaching approaches for ES in non-clinical and clinical environments
- Existing barriers that complicate embedding ES in the curriculum
- Existing drivers that support embedding ES in the curriculum.

The survey was tested for face and content validity with a group of five academics from three dental schools in the UK and ROI. The group were asked to access and complete the questionnaire via the online platform and provide comments via email. A final draft version of the questionnaire was developed considering the responses to improve clarity of language and ease of understanding.

The intended respondents were deans, heads of schools, directors of education, or senior academics of all dental schools in the UK and ROI that deliver undergraduate dentistry/dental surgery programmes, as recognised by the General Dental Council of the UK and the Irish Dental Council. At the time of the survey, this was 18 dental schools. The survey was supported and distributed by the Dental Schools Council to dental schools in the UK and ROI. It was requested that the deans, heads of schools, or directors of education complete the survey where possible, unless they identified a designated individual that was best placed to answer the questions (eg school sustainability champion, senior academics in strategic positions). In addition to this recruitment stream, the authors directly contacted the institutions that did not respond to Dental School Council request on one further occasion. No further reminders were sent. All responses were anonymous, no personal data was collected and informed consent was gained at the start of the survey through the provision of a participant information statement and consent form.

The close-ended questions were analysed through descriptive statistics and were presented in tables and charts. The free-text responses were analysed through content analysis, where responses were coded, quantified and presented in tables to illustrate frequency of responses.

## Results

In total, 18 dental schools completed the survey from October 2023 to January 2024, representing a 100% response rate from the intended respondents. One school provided two different responses, potentially due to delegation or overlap from the initial invitation email and the follow-up. The two responses were collated on the master spreadsheet to enable a single response from the school and to avoid omitting key information from two different responders. For the double response, the open-ended responses were identical and the barriers and free-text responses were combined; no data was deleted.

### Current teaching approaches for ES in non-clinical and clinical environments

**Fig. 1:**
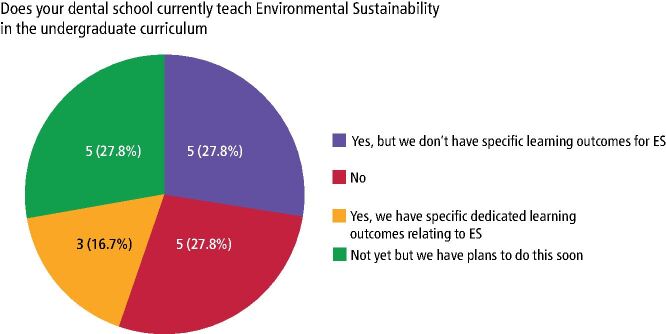
A pie chart demonstrating current teaching of ES in the undergraduate curriculum in the UK and ROI

Ten dental schools (55.6%) reported that they do not currently teach ES; although, five of these schools (27.8%) plan to do this soon (Fig. 1). Five schools (27.8%) currently teach ES without dedicated learning outcomes and three schools (16.7%) have specific learning outcomes for ES.

**Fig. 2:**
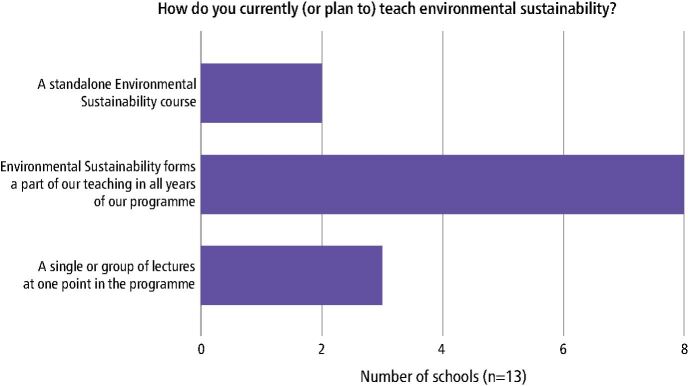
A bar chart demonstrating current or planned approaches to teaching ES in the undergraduate curriculum

Of the 13 schools that currently teach or have plans to teach ES, over 60% (n = 8) of respondents reported that ES should form a part of teaching in all years of undergraduate dental programmes (Fig. 2). From the remaining schools, three deliver a single or groups of lectures at one point in the programme and two schools propose a standalone ES course.

Lectures (n = 7) were the most reported method to teach ES in non-clinical environments (Table 2). Smaller group teaching in the form of tutorials or workshops (n = 4) were also reported by multiple dental schools. At the time of completion of the survey, four dental schools were finalising their delivery method for ES in non-clinical environments and five schools reported the same for clinical settings (Table 3). Three schools reported to focusing teaching on the environmental benefits of delivering high-quality oral healthcare as a strategy for teaching ES in clinical spaces.

**Table 2:** Free-text responses to current or planned learning and teaching methods for ES in non-clinical environments

Learning and teaching method (non-clinical)	Number of responses
Lectures	7
Tutorials/workshops	4
Still finalising delivery method/unsure/in discussion	4
Online module/training package	2
Problem-based learning	2
Self-directed learning/projects	2
Embedded into all teaching events where appropriate	1
Culture change (eg shared spaces)	1
Plenaries/teaching event briefing	1

**Table 3:** Free-text responses to current or planned learning and teaching methods for ES in clinical environments

Learning and teaching method (clinical)	Number of responses
Still finalising delivery method/unsure/in discussion	5
Focus on high-quality oral healthcare delivery	3
Mentioned in clinical skills laboratory induction	1
Raising awareness on clinics	1
Opportunistic student initiatives	1
Problem-based learning	1
Using sustainable materials/instruments in clinic	1
Critique existing practices	1
Adopt digital workflows	1
Small group clinical teaching	1

### Existing barriers that complicate embedding ES in the curriculum

**Fig. 3:**
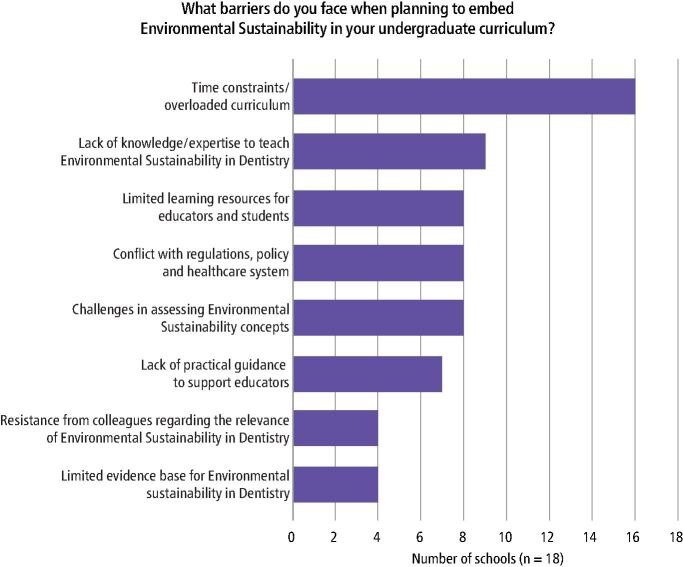
A bar chart demonstrating the frequency of reported barriers that complicate embedding ES in the undergraduate curriculum

Almost 90% of dental schools (n = 16) reported time constraints and the overloaded curriculum as a major barrier to embedding ES in the undergraduate curriculum (Fig. 3). A lack of knowledge/expertise to teach ES in dentistry (n = 9), limited availability of learning resources for educators and students (n = 8), conflict with regulations, policy and healthcare systems (n = 8), challenges in assessing ES concepts (n = 8) and a lack of practical guidance to support educators in embedding ES (n = 7) were also commonly identified barriers.

Additional barriers identified through free-text responses were cost (n = 3), awaiting publication of an updated national curriculum framework (n = 1) and the absence of an ES strategy from the associated hospital trust (n = 1).

### Existing drivers that support embedding ES in the curriculum

**Fig. 4:**
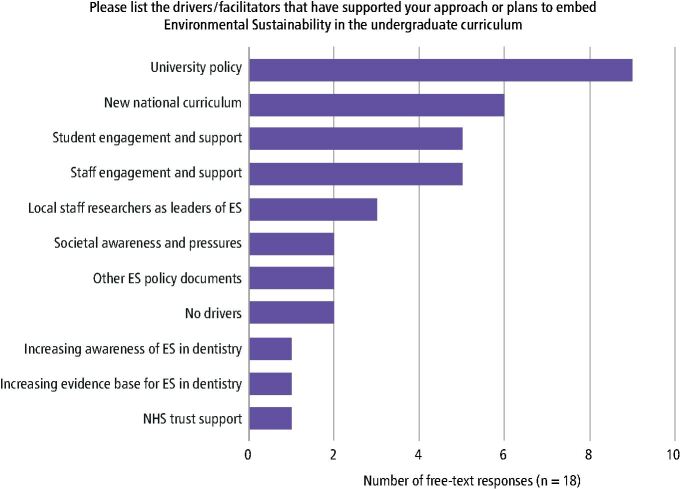
A bar chart demonstrating the free-text responses to the drivers/facilitators that support the incorporation of ES in the undergraduate curriculum

The most reported driver supporting embedding ES in the curriculum was the influence of local higher education institutions (n = 9) and the incorporation of ES into university strategies and policies (Fig. 4). The incorporation of ES into the national curriculum (n = 6), student (n = 5) and staff (n = 5) engagement and support, and local staff researchers as leaders of ES (n = 3), were also commonly reported drivers.

## Discussion

This study aimed to report current practice, barriers and drivers to embedding ES in the undergraduate dental curriculum in the UK and ROI. The incorporation of ES learning outcomes into the General Dental Council's *The safe practitioner* framework mandates teaching and assessment of this topic from September 2025. The date of implementation for these changes is proving to be a real challenge, as over 50% of dental schools have not yet included ES in their local curriculum.

### Current teaching approaches for ES in non-clinical and clinical environments

Most schools perceive that embedding ES into all years of the dental programme is the most appropriate curricular approach. This aligns with the recommendations set out by the ADEE consensus documents on ES.^8^^,^^9^ The attitudinal or value-based learning required for ES is similar to topics such as professionalism, which is already embedded into curricula in a longitudinal manner, with vertical and horizontal integration.^21^^,^^22^ Standalone courses or single events at one point in the curriculum may be feasible in some institutions but it is unlikely to promote longitudinal learning of complex constructs like ES. Achieving meaningful changes in attitudes and behaviours is more likely to result from incremental learning that builds from simple to more sophisticated messages over time.

Dental schools appear better prepared to teach ES in non-clinical environments, with multiple methods reported in this survey. Didactic approaches, including lectures and tutorials, are common approaches to information delivery, and these are favoured for ES. This works particularly well to establish a baseline of fundamental knowledge in the early years for planetary health and ES in healthcare. Recent research by Dixon *et al*.^23^ provides evidence-based and subject-specific content statements that can be directly incorporated into teaching events. These statements were mapped to all curriculum subjects in dentistry, allowing educators from all disciplines to adopt these statements into their course material and teaching modalities. This approach should allow easy implementation by embedding short statements into existing lectures and allows frequent revisiting of the topic across all years of the programme. An incremental approach should embed the message that ES is a core part of oral healthcare delivery.

The proposed teaching methods for ES in clinical environments are less clear, with most responses demonstrating that schools are yet to finalise their planned approaches. In many respects, this imbalance is expected, as it is widely acknowledged that there are numerous barriers to delivering environmentally sustainable oral healthcare in the current climate.^4^^,^^24^^,^^25^^,^^26^^,^^27^ While these undoubtedly complicate the delivery of environmentally sustainable oral healthcare, it is important to be aware that the environmental impacts of oral healthcare can be mitigated through the provision of high-quality oral healthcare.^11^ Reducing the need to treat through preventive care, providing high-quality operative care that limits repairs and replacement, integrated care with smart structuring and organisation of patient care, and professional and patient ownership of care, all mitigate environmental impacts.^11^^,^^28^ In addition to making an immediate impact to mitigate environmental impacts, educators and institutions have a role to develop the future workforce who should engage with stakeholders and challenge current unsustainable practices.^23^

### Existing barriers that complicate embedding ES in the curriculum

The answer options for this question were developed from a comprehensive literature search of the health professional literature, but a free-text option was incorporated to allow full exploration of all barriers facing UK and Irish dental schools. The responses to this survey demonstrate that educators experience numerous barriers that complicate embedding ES in the curriculum. A lack of space and time in the curriculum is a widely reported issue in all health professional programmes and this complicates the incorporation of ‘additional' ES content. It is argued that careful and strategic incorporation of ES into existing teaching events across all years of programmes will reduce the need to ‘add' new events.^9^ Although, good academic practice and governance should ensure that a curriculum remains valid and is sustainable through an approach of review and revision in a development cycle that should be in place to remove irrelevant and outdated components.

A lack of knowledge and expertise regarding ES and limited resources for educators and students were frequently reported barriers in this study. The availability of learning resources at all levels is increasing, namely with the FDI World Dental Federation's MOOC (bit.ly/FDI-ES-MOOC), ADEE's learning outcomes and methods of teaching and assessment (bit.ly/ADEE-ES-LO) and the recent evidence-based subject-specific content guidelines for ES (bit.ly/ES-CONTENT).^9^^,^^12^^,^^23^ These are all available as open access and are designed to support educators and students at all levels (Table 4). It is envisaged that these core documents will promote ES and provide a platform for further development of additional resources.

**Table 4:** Open-access resources and links

Open-access resources for environmental sustainability in dentistry and oral healthcare	Link
FDI World Dental Federation's MOOC	bit.ly/FDI-ES-MOOC
ADEE consensus report on learning outcomes and methods of teaching and assessment for environmental sustainability	bit.ly/ADEE-ES-LO
Evidence-based, subject-specific content statements for environmental sustainability	bit.ly/ES-CONTENT

Clinical regulations are acknowledged as a common barrier, particularly in relation to cross-infection control. Duane *et al*.^24^outlined many of these challenges in a critical review of the HTM 01-05 policy document. Strategies to teach ES in clinical environments were discussed above and additional methods were outlined in the ADEE consensus statement.^9^

The challenge of assessing ES is a common concern among academics. While assessment of environmentally sustainable behaviours in the provision of oral healthcare may be complex at present, assessment of student awareness, attitudes and knowledge should be achievable with existing methods. Multiple assessment methods have been recommended for ES in dentistry.^9^ Similar to embedding content into existing teaching events, there are multiple opportunities to augment or modify existing assessment methods to incorporate ES, which may include awarding marks in an objective structured clinical examination, written papers, or within clinical grading.^23^

### Existing drivers that support embedding ES in the curriculum

A positive finding is that universities are the most common driving force to support curriculum development. Increasingly, ES is forming a key part of university strategies and many institutions are demanding ES to be taught across all programmes. The recent publication of *The safe practitioner* framework by the UK national regulator of professional standards, the General Dental Council, is also a major driver for this change. Staff and student support for change has been recognised through previous surveys from across the world.^13^^,^^14^^,^^15^^,^^16^

## Conclusion

Currently, ES is not taught in most dental schools in the UK and ROI. While the learning outcomes detailed in the General Dental Council's *The safe practitioner* framework will mandate this change, many schools face challenges in finalising the delivery modalities of ES teaching, particularly in clinical environments. Numerous barriers have been identified that complicate embedding ES in the curriculum, namely the overloaded curriculum and a lack of expertise and resources. Positively, universities, staff, students and the recently implemented learning outcomes are driving impactful change across the sector.

## Data Availability

The data supporting this study's findings are openly available in the University of Sheffield Research Data Repository at https://doi.org/10.15131/shef.data.26506234 under the terms of the Creative Commons Attribution (CC BY-NC 4.0) licence.
